# Assessment of Radiation Doses Delivered to Organs at Risk Among Patients With Early-Stage Favorable Hodgkin Lymphoma Treated With Contemporary Radiation Therapy

**DOI:** 10.1001/jamanetworkopen.2020.13935

**Published:** 2020-09-29

**Authors:** Chelsea C. Pinnix, Jillian R. Gunther, Penny Fang, Mikaela E Bankston, Sarah A. Milgrom, David Boyce, Hun Ju Lee, Ranjit Nair, Raphael Steiner, Paolo Strati, Sairah Ahmed, Swaminathan P. Iyer, Jason Westin, Simrit Parmar, M. Alma Rodriguez, Loretta Nastoupil, Sattva Neelapu, Christopher Flowers, Bouthaina S. Dabaja

**Affiliations:** 1 Department of Radiation Oncology, The University of Texas MD Anderson Cancer Center, Houston; 2 Department of Radiation Oncology, University of Colorado, Denver; 3 Department of Lymphoma/Myeloma, The University of Texas MD Anderson Cancer Center, Houston

## Abstract

**Importance:**

Response-adapted randomized trials have used positron emission tomography–computed tomography to attempt to identify patients with early-stage favorable Hodgkin lymphoma (ESFHL) who could be treated with doxorubicin, bleomycin, vinblastine, and dacarbazine (ABVD) without radiation therapy (RT). While maximal efficacy is demonstrated with combined modality therapy, RT is often omitted in fear of late adverse effects; however, the application of modern RT could limit these toxic effects.

**Objective:**

To determine the radiation doses delivered to organs at risk with modern involved-site RT among patients with ESFHL treated with 20 Gy after 2 cycles of ABVD.

**Design, Setting, and Participants:**

This case series included 42 adult patients with ESFHL (according to the German Hodgkin Study Group criteria) who were treated between 2010 and 2019, achieved complete response by positron emission tomography–computed tomography (1-3 on 5-point scale) following 2 cycles of ABVD, and then received consolidative RT. The study was conducted at a single comprehensive cancer center.

**Exposures:**

2 cycles of chemotherapy followed by 20-Gy involved-site RT.

**Main Outcomes and Measures:**

The medical records of patients with ESFHL were examined. Organs at risk were contoured, and doses were calculated. Progression-free survival, defined from date of diagnosis to disease progression, relapse, or death, and overall survival were estimated using the Kaplan-Meier method.

**Results:**

The cohort comprised 42 patients with ESFHL (median [range] age at diagnosis, 35 [18-74] years; 18 [43%] women; 24 [57%] with stage II disease). At a median follow-up of 44.6 (95% CI, 27.6-61.6) months, the 3-year progression-free survival and overall survival rates were 91.2% (95% CI, 74.9%-97.1%) and 97.0% (95% CI, 80.4%-99.6%), respectively. The mean heart dose was less than 5 Gy (mean, 0.8 Gy; SD, 1.5 Gy; range, 0-4.8 Gy) in all patients. The mean (SD) breast dose for both breasts was 0.1 (0.2) Gy (left breast range, 0-1.0 Gy; right breast range, 0-0.9 Gy).

**Conclusions and Relevance:**

In this study, combined modality therapy with 2 cycles of ABVD and 20 Gy for ESFHL was highly effective and avoided excess doses to organs at risk, which may limit long-term toxic effects.

## Key Points

**Question:**

What are the radiation doses to normal tissues for patients with favorable Hodgkin lymphoma treated with modern involved-site radiation therapy?

**Findings:**

This case series study included 42 patients with early-stage favorable Hodgkin lymphoma who were treated with combined modality therapy. The mean heart dose was less than 5 Gy in all patients, and the mean breast dose was 1 Gy or less in all 18 women.

**Meaning:**

In this study, combined modality therapy for favorable Hodgkin lymphoma with contemporary radiation therapy delivered low radiation doses to organs at risk.

## Teaser

This case series study investigates the radiation doses delivered to organs at risk with modern involved-site radiation therapy among patients with early-stage favorable Hodgkin lymphoma treated with 20 Gy after 2 cycles of doxorubicin, bleomycin, vinblastine, and dacarbazine (ABVD).

## Introduction

The HD.6 trial, which compared outcomes among patients with limited-stage Hodgkin lymphoma (HL) who were treated with doxorubicin, bleomycin, vinblastine, and dacarbazine (ABVD) alone vs subtotal nodal radiation therapy (RT) with or without 2 cycles of ABVD, demonstrated superior 12-year progression-free survival (PFS) with combined modality therapy (CMT) but inferior 12-year overall survival (OS) rates, which were attributed to increased cardiovascular events and secondary malignant neoplasms.^1^ This trial is cited as a cautionary tale for oncologists who recommend CMT; however, it is not reflective of today’s treatment approaches, which incorporate involved-site radiation therapy (ISRT) delivered with modern techniques.

Treatment of HL is a major success of modern oncology. With favorable disease outcomes comes the need to balance efficacy and the potential toxic effects of curative therapy.^2^ Recent trials have focused on identifying patients at a lower risk of relapse, representing a patient population for whom treatment de-escalation would be appropriate.^3^ The German Hodgkin Study Group (GHSG) identified factors that are prognostic among patients with limited-stage HL.^4,5^ Patients with stage I or stage II disease without risk factors (ie, extranodal lesions, bulky mediastinal involvement, ≥3 involved nodal areas, and erythrocyte sedimentation rate of ≥30 with B symptoms or of ≥50 without B symptoms) are considered to have early-stage favorable HL (ESFHL) and have excellent outcomes after 2 cycles of ABVD followed by 20 Gy of consolidative involved-field RT, based on the GHSG HD10 trial.^5^ In the follow-up GHSG HD16 study, 2 cycles of ABVD and RT to 20 Gy was compared with ^18^F-fluorodeoxyglucose positron emission tomography–computed tomography (PET-CT)–guided therapy with omission of RT after a negative PET-CT scan.^6^ Recently, other response-adapted HL studies have used PET-CT imaging to identify patients who could be treated with abbreviated ABVD without consolidative RT.^7,8^ While these studies have demonstrated maximal disease control for patients treated with CMT, concerns regarding potential long-term radiation-related toxic effects, including secondary malignant neoplasms and cardiac damage, have prompted some oncologists to offer ABVD alone as an alternative treatment strategy.^9^

Compared with involved-field RT and more historic subtotal nodal and mantle fields, modern ISRT offers a significant RT field reduction.^10^ Patterns of failure among patients with limited-stage HL indicate that the dominant pattern of relapse after ABVD alone is in previously involved lymph nodes, providing a basis for ISRT.^6,7^ With this approach, the RT field is limited to sites of disease at diagnosis without prophylactic targeting of nodal regions that did not demonstrate evidence of disease on baseline PET-CT imaging. The effect of this RT field size reduction would be expected to be substantial on patients with ESFHL who have low-volume, nonbulky disease burden limited to 2 or fewer regions.

We sought to evaluate outcomes of patients with ESFHL who were treated with CMT according to the control arm of the GHSG HD16 trial. Given the recognized long latency period of RT-induced adverse effects, coupled with the relatively short follow-up of patients treated with ISRT, we were unable to use the incidence of late effects as an end point. Therefore, we performed a dosimetric analysis to determine the radiation doses received by normal structures as a surrogate for possible late toxic effects. Hypothesizing that treatment with modern ISRT may result in reduced RT exposure to normal tissues, we collected data regarding doses to organs at risk (OARs) with ISRT among patients treated with 20 Gy after 2 cycles of ABVD.

## Methods

We examined the medical records of HL patients aged 18 years and older who were treated with CMT at MD Anderson Cancer Center (Houston, USA) after approval by the MD Anderson Cancer Center institutional review board. A waiver of informed consent was granted by our institutional review board in accordance with the Declaration of Helsinki.^11^ We screened our radiation oncology database for patients with HL who were treated with a dose of 20 Gy between 2010 and 2019. Patients with ESFHL, as defined by the GHSG, who achieved a complete response according to PET-CT imaging (1-3 on a 5-point scale, per Lugano criteria) after 2 cycles of chemotherapy and went on to receive 20 Gy of consolidative ISRT were eligible for inclusion in the retrospective analysis.^5,12^ Cases were excluded if patients had ESFHL and were treated to 20 Gy but received 3 cycles of ABVD (2 patients) or 4 cycles of AVBD (5 patients).

Consolidative 20 Gy RT was administered with clinical target volume (CTV) delineation, based on the International Lymphoma Radiation Oncology group ISRT guidelines.^10^ Deep inspiration breath-hold was used for most patients treated to the mediastinum.^10^ All women were treated on an inclined board for maximal breast sparing.^13^ Intensity-modulated radiation therapy and volumetric-modulated arc therapy were used at the discretion of the treating physician. OARs were contoured, including the total heart, total lungs, bilateral breasts (in female patients), thyroid gland, and salivary glands (bilateral parotid and submandibular glands).^14^ The takeoff of the left main coronary artery was used to characterize disease involvement limited to above the heart.^15^ Dosimetric constraints for patients with hematologic malignant neoplasms were used to guide treatment planning. OAR planning goals included mean heart dose (MHD) of 5 Gy or less,^16,17^ mean lung dose of 13.5 Gy or less,^18^ volume of the total lung that received 5 Gy (V5) of 55% or less,^18^ mean breast dose of less than 4 Gy,^19^ and volume of the thyroid gland that received 20 Gy (V20) or less than 86%.^20^ For patients treated with 20 Gy, in which RT plans would be expected to easily achieve these constraints, the principle of as low as reasonably achievable (ALARA) was used. The volume of the clinical target volume in cubic centimeters (cc) was recorded. Toxic effects were graded per Common Terminology Criteria for Adverse Events version 5.

### Statistical Analysis

PFS was defined from date of diagnosis to disease progression, relapse, or death from any cause. OS was defined from date of diagnosis to death. PFS and OS were estimated using the Kaplan-Meier method.^21^ Median follow-up time and 95% CIs were estimated using the reverse Kaplan-Meier method.^22^ We do not report any data that used *t* tests in this analysis, and no comparisons were performed, so no prespecified level of significance was set. Statistical analyses were performed using Prism version 8.0 (GraphPad) and SPSS statistical software version 24 (IBM Corp).

## Results

Between 2010 and 2019, 42 patients with ESFHL received CMT (Table). The median (range) age was 35 (18-74) years; 18 patients (43%) were women, and 24 (57%) had stage II disease. A total of 25 patients (60%) had 2 nodal regions of involvement, and 4 patients (10%) presented with B symptoms. The most common disease presentation was unilateral neck involvement (15 [36%]), followed by unilateral neck and mediastinal involvement (11 [26%]). The mediastinum was involved in 14 patients (33%). Among these patients, 11 (79%) had disease that extended below the carina, and 2 (14%) had disease that extended below the left main coronary artery.

**Table.** Clinical and Treatment Characteristics of 42 Patients Treated With Combined Modality Therapy for Early-Stage Favorable Hodgkin Lymphoma

Characteristic	Patients, No. (%) (N = 42)
Age at diagnosis, median (range), y	35 (18-74)
Women	18 (43)
Disease stage	
I A	16 (38)
I B	2 (5)
II A	22 (52)
II B	2 (5)
Mediastinum involvement	14 (33)
Below the carina^a^	11 (79)
Below the left main coronary artery^a^	2 (14)
Nodal regions involved, No.^b^	
1	17 (40)
2	25 (60)
Sites of involvement	
Unilateral neck	15 (36)
Bilateral neck	7 (17)
Unilateral neck and mediastinum	11 (26)
Unilateral neck and axilla	2 (5)
Unilateral axilla	1 (2)
Isolated mediastinum	3 (7)
Unilateral groin	1 (2)
Unilateral groin and pelvis	1 (2)
Unilateral epitrochlear	1 (2)
B Symptoms	4 (10)
Histology	
Nodular sclerosing	22 (52)
Lymphocyte rich	7 (17)
Mixed cellularity	13 (31)
Chemotherapy type	
ABVD	36 (86)
AVD	5 (12)
ABD	1 (2)
Cycles, No.	2
RT Modality	
IMRT or VMAT	36 (86)
3DCRT	5 (12)
Protons	1 (2)
Deep inspiration breath-hold^a^	9 (64)
Clinical target volume, mean (SD) [range], cc	201.7 (165.5) [32.6-859.3]

Abbreviations: 3DCRT, 3-dimensional conformal radiation therapy; ABD, doxorubicin, bleomycin, and dacarbazine; ABVD, doxorubicin, bleomycin, vinblastine, dacarbazine; AVD, doxorubicin, vinblastine, dacarbazine; IMRT, intensity-modulated radiation therapy; RT, radiation therapy; VMAT, volumetric-modulated arc therapy.

^a^ Percentage based on the 14 patients with mediastinal involvement.

^b^ Nodal regions are defined according to the German Hodgkin Study Group.

Most patients (36 [86%]) were treated with 2 cycles of ABVD; 5 patients (12%) received doxorubicin, vinblastine, dacarbazine; 1 patient (2%) received doxorubicin, bleomycin and dacarbazine. RT was administered with intensity-modulated radiation therapy or volumetric-modulated arc therapy in 36 patients (86%); proton therapy was administered to 1 patient (2%). The mean (SD) CTV was 201.7 (165.5) cc (range, 32.6-859.3 cc).

At a median follow up of 44.6 (95% CI, 27.6-61.6) months, the 3-year PFS was 91.2% (95% CI, 74.9%-97.1%) and OS was 97.0% (95% CI, 80.4%-99.6%). The median PFS and OS were not reached. Two relapses occurred; 1 patient relapsed 2.1 years after RT, in field, at an initial disease site, and the second relapse occurred 3.5 months after RT outside of the field. A patient in their 70s died, unrelated to HL.

Overall treatment was well tolerated with no grade 4 or 5 Common Terminology Criteria for Adverse Events toxic effects. Only 1 grade 3 Common Terminology Criteria for Adverse Events toxic effect occurred in a patient who experienced a myocardial infarction after the first cycle of ABVD. No secondary malignant neoplasms occurred in the follow-up period.

**Figure 1.**
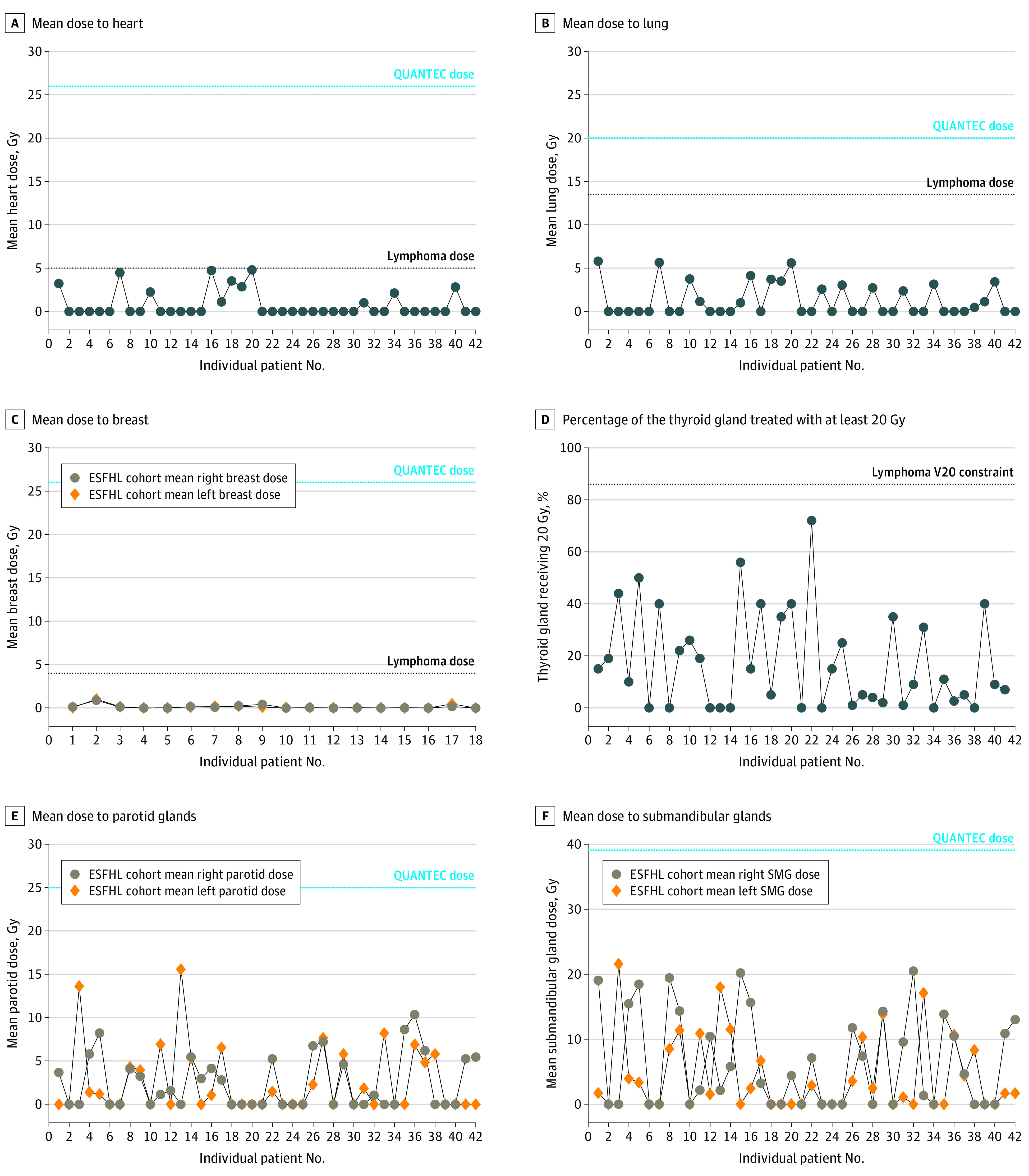
Radiation Doses Delivered to Normal Tissues Among 42 Patients With Early-Stage Favorable Hodgkin Lymphoma (ESFHL) Treated With Combined Modality Therapy QUANTEC indicates Quantitative Analysis of Normal Tissue Effects in the Clinic; V20, the percentage of the thyroid gland treated with at least 20 Gy.

**Figure 2.**
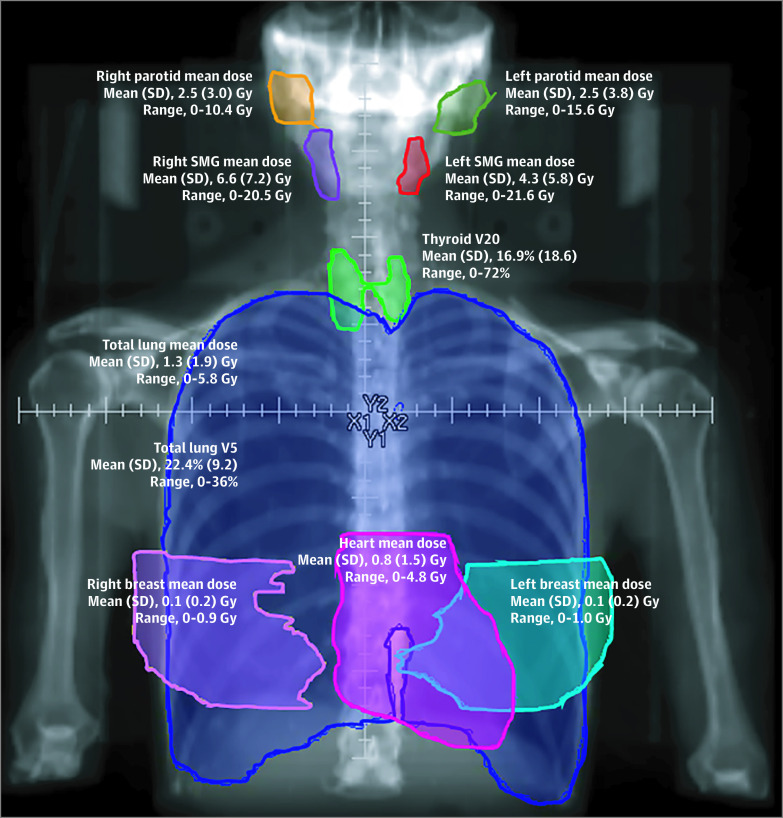
Schematic of Doses to Normal Tissues

Standard radiation dose constraints for various normal tissues were established by a comprehensive review of the association of radiation dose and volume with organ function and toxic effects (Quantitative Analysis of Normal Tissue Effects in the Clinic [QUANTEC]).^23^ These dose constraints are often appropriate for patients with solid tumors, for whom higher RT doses are used. For patients with lymphoma, much lower RT organ constraints are considered appropriate, as established by survivorship studies and/or dosimetric RT studies based on patient populations with lymphoma.^16,17,18,19,20,24^ We compared the RT doses administered to the heart (Figure 1A), lung (Figure 1B), breasts among the 18 female patients (Figure 1C), thyroid gland (Figure 1D), parotid glands (Figure 1E), and submandibular glands (Figure 1F) with the QUANTEC and lymphoma dose constraints. The mean heart mean dose was 0.8 Gy (range, 0-4.8 Gy) (Figure 2); all patients were below both QUANTEC (26 Gy) and lymphoma (5 Gy) MHD constraints. The mean (SD) MHD for 12 patients with mediastinal disease above the left main coronary artery was 1.8 (1.7) Gy (range, 0-4.5 Gy), while the mean (SD) MHD for 2 patients with mediastinal involvement below the left main coronary artery was 4.8 (0.04) Gy (range, 4.7-4.8 Gy). Among the 18 female patients, the mean (SD) right and left breast dose was 0.1 (0.2) Gy, well below the 4 Gy lymphoma constraint associated with increased risk of secondary breast cancer.^19^ All patients achieved the lung lymphoma dose constraints of a mean lung dose of less than 13.5 Gy and total lung that received less than 5 Gy of 55% (Figure 2).

RT-related hypothyroidism is associated with the volume of the thyroid gland that receives radiation. The volume of the thyroid gland treated to 25 and 30 Gy have been identified as the strongest factors associated with radiation-induced hypothyroidism among patients with HL.^20,24^ However, for patients treated with RT doses of less than 30 Gy, other constraints are relevant. It has been shown that when the volume of the thyroid gland treated to 20 Gy exceeds 86%, the incidence of hypothyroidism is greater than 80%.^20^ No patient exceeded this constraint. The mean doses to the salivary glands were low and well below the standard constraints of 26 Gy and 39 Gy (for parotid and submandibular glands, respectively), suggesting minimal risk for long-term xerostomia.^23^

## Discussion

This study confirms the excellent outcome among patients with ESFHL treated with CMT. In our study, the 3-year PFS was 91.2% with 2 relapses occurring in the follow-up period. There were no deaths owing to disease. To date, toxic effects were limited. Radiation doses to OARs were exceedingly low, including the heart (<5 Gy in all patients) and the breasts (≤1 Gy for all female patients).

The EORTC H10F and UK Rapid trials were 2 noninferiority randomized clinical trials designed to assess the effect of RT omission among patients who achieved a PET-CT complete response after 2 or 3 cycles of ABVD, respectively.^7,8^ Both studies showed inferior PFS among patients who did not receive RT, despite a negative interim PET-CT scan. In the GHSG HD16 trial, among 628 patients with PET-2 negative scans, the 5-year PFS was 93.4% with CMT and 86.1% with ABVD alone.^6^ Taken together, these studies highlight the loss of tumor control with the omission of RT, even among the lowest-risk group.

The argument against RT for patients with ESFHL is weighted in frightening historic data on the risks of cardiac disease and secondary malignant neoplasms after CMT. Given the long latency period (>10 years) between treatment and the development of these potential adverse effects, the mature data that exist are based on older radiation techniques, higher RT doses, and significantly larger fields.^25,26^ In a series of roughly 4000 patients who survived HL, the cumulative 30-year incidence of any second cancer (including myelodysplastic syndrome) was 33.2% compared with the expected incidence of 9.6%.^27^ The most common solid secondary malignant neoplasms were female breast cancer and lung cancer (30-year incidence of 16.6% and 7.1%, respectively). Many patients were treated with historic planning techniques, 2-dimensional treatment planning (as opposed to CT-based 3-dimensional treatment planning), doses of 40 Gy, and mantle-field RT, with (30.1%) or without (20.9%) infradiaphragmatic RT. Given these alarming rates of secondary cancers, it is not surprising that many oncologists would elect to forgo the potential 4% to 12% benefit in PFS in exchange for the mitigation of secondary malignant neoplasm risk. However, for patients with ESFHL (who by definition have limited regions of disease involvement), lower doses of 20 Gy, ISRT volumes, and the use of modern radiotherapy with contemporary treatment techniques can result in reduced normal tissue exposure. We routinely use a 15° incline board for patient setup during treatment to permit inferior displacement of breast tissue out of the RT field.^13^ Avoidance of lateral beams in anterior-posterior weighted (ie, butterfly) intensity-modulated radiation therapy and volumetric-modulated arc therapy approaches can reduce lung and breast RT dose.^28,29^ Deep inspiration breath-hold decreases pulmonary and cardiac exposure.^30,31^ With these techniques, the mean right and left breast dose was 1 Gy or less in every patient; the average mean lung dose was only 1.3 Gy. While historic data are illustrative regarding the harmful late effects of RT, current dosimetric data suggests these treatment modifications will result in significant reductions in late second cancer risk.^32^

Cardiac morbidity and mortality are other significant considerations among patients with HL. In the Childhood Cancer Survivor Study of 14 358 five-year cancer survivors, mean heart radiation doses of greater than 15 Gy increased the relative hazard of cardiac events by 2-fold to 6-fold compared with survivors who did not receive RT.^16^ An additional study of European childhood cancer survivors treated before 1986 found increased cardiac mortality risk with a MHD of greater than 5 Gy.^17^ Given that many survivorship studies include patients treated with 2-dimensional RT, there is limited long-term data regarding cardiac toxic effects with detailed information of the doses delivered to the heart. In a case-control study nested in a cohort of 2617 five-year survivors of HL diagnosed between 1965 and 1995, MHDs were estimated via reconstruction of 2-dimensional RT plans.^33^ Among the 91 patients who experienced heart failure as an initial cardiovascular diagnosis compared with 278 matched controls, the median MHD for the group was 25.8 Gy. The MHD was at least 21 Gy in most cases (69%). The number of cases with an MHD of less than 5 Gy were not reported. In another nested case-control study based on the same population of 2617 survivors of HL with reconstruction of 2-dimensional RT plans, 325 patients who experienced coronary heart disease as a first cardiovascular event after HL treatment were compared with 1204 matched controls.^34^ Overall, 9% of the 325 cases had an MHD of 5 Gy or less, and 21% of the controls had an MHD of 5 Gy or less. The rate ratio for coronary heart disease was 1.0 for the 5% of cases that had an MHD of 0 and 1.19 for the 4% of cases that had a MHD of 1 to 5 Gy. Neither rate ratio was statistically significant. It is unclear what proportion of the patients in these studies had ESFHL. In the current study of patients with ESFHL, the MHDs were below 5 Gy in every case. Among the 14 patients with mediastinal involvement, 86% had disease above the heart. We suggest that the risk of long-term cardiac morbidity should be assessed on a case-by-case basis. For patients for whom the MHD from RT would be expected to be less than 5 Gy, consideration must be given to the potential cardiac risk of additional cycles of ABVD alone, which will result in increased doxorubicin administration. RT plans with low MHDs could actually confer less cardiac risk than extra cycles of doxorubicin,^16^ if additional ABVD was planned to compensate for RT omission.

Our study highlights the ability to achieve very low OAR doses for patients with ESFHL with thoughtful radiation treatment planning; therefore, dose constraints developed for solid tumor treatments may be excessive and harmful. For the entire cohort of patients with favorable HL treated at 20 Gy, attainment of lower lymphoma dose constraints was easily achievable with ISRT and modern technology. As a result, it is our institutional practice to optimize RT plans well below the allowable lymphoma dose constraint using the ALARA principle. We frequently optimize and evaluate the volume of normal tissue that receives low doses of 5 Gy over the course of the entire radiation course in an effort to leave as much normal tissue untouched by radiation as possible.

### Limitations

This study has limitations, including the small cohort size. Moreover, the data are only from a single cancer center, and pediatric cases were not included. Importantly, the short follow-up time does not permit determination of long-term risk of toxic effects; therefore, we are unable to correlate reduced OAR RT exposure with decreased incidence of late morbidity and mortality. Future studies of patients with HL who are treated with CMT should include detailed dosimetric information regarding RT doses to OARs to help to quantify and understand long-term toxic effects risk.

## Conclusions

The findings of this study indicate that 2 cycles of ABVD followed by 20 Gy RT is an effective strategy for the management of ESFHL. The favorable dosimetric profiles, with mean doses of less than 5 Gy to surrounding normal structures, suggests that long-term toxic effects may be lower than what has been reported in historic series. We advise that treatment strategies for patients with ESFHL, including the incorporation of RT, be considered on a case-by-case basis.
